# Exploring the Interaction Mechanism Between Cyclopeptide DC3 and Androgen Receptor Using Molecular Dynamics Simulations and Free Energy Calculations

**DOI:** 10.3389/fchem.2018.00119

**Published:** 2018-04-19

**Authors:** Huimin Zhang, Tianqing Song, Yizhao Yang, Chenggong Fu, Jiazhong Li

**Affiliations:** ^1^School of Pharmacy, Lanzhou University, Lanzhou, China; ^2^College of Chemistry and Chemical Engineering, Lanzhou University, Lanzhou, China

**Keywords:** Cyclopeptide DC3, androgen receptor, protein drug interaction, homology modeling, molecular docking, molecular dynamics simulations

## Abstract

Androgen receptor (AR) is a key target in the discovery of anti-PCa (Prostate Cancer) drugs. Recently, a novel cyclopeptide *Diffusa Cyclotide-3* (DC3), isolated from *Hedyotisdiffusa*, has been experimentally demonstrated to inhibit the survival and growth of LNCap cells, which typically express T877A-mutated AR, the most frequently detected point mutation of AR in castration-resistant prostate cancer (CRPC). But the interaction mechanism between DC3 and AR is not clear. Here in this study we aim to explore the possible binding mode of DC3 to T877A-mutated AR from molecular perspective. Firstly, homology modeling was employed to construct the three-dimensional structure of the cyclopeptide DC3 using 2kux.1.A as the template. Then molecular docking, molecular dynamics (MD) simulations, and molecular mechanics/generalized Born surface area (MM-GBSA) methods were performed to determine the bind site and explore the detailed interaction mechanism of DC3-AR complex. The obtained results suggested that the site formed by H11, loop888-893, and H12 (site 2) was the most possible position of DC3 binding to AR. Besides, hydrogen bonds, hydrophobic, and electrostatic interactions play dominant roles in the recognition and combination of DC3-AR complex. The essential residues dominant in each interaction were specifically revealed. This work facilitates our understanding of the interaction mechanism of DC3 binding to AR at the molecular level and contributes to the rational cyclopeptide drug design for prostate cancer.

## Introduction

Prostate cancer (PCa) has become the second frequently diagnosed cancer in men throughout the world (American Cancer Society, 2015). Prostate, lung and bronchus, colorectal cancers accounts for about 44% of all cancer cases in men, with PCa alone accounting for 1 in 5 new diagnoses (Siegel et al., 2016). PCa is especially common in economically developed countries and regions like Northern and Western Europe, Northern America, and Oceania (American Cancer Society, 2015). In America, prostate cancer is the most common cancer and was predicted as the leading cause of male cancer-related death over the next decade (Siegel et al., 2016). In those less developed countries, the incidence rate of prostate cancer is increasing with stable or increasing mortality trend in recent years (Center et al., 2012).

Androgen receptor (AR) (NR3C4, nuclear receptor subfamily 3, group C, gene 4), a member of steroid hormone group of nuclear receptor superfamily, plays an essential role in the development and proliferation of prostate cancer (Tsai and O'Malley, 1994; Mangelsdorf et al., 1995; Nuclear Receptors Nomenclature Committee, 1999). The survival and growth of PCa cells are dependent on the androgenic stimulation through AR. Firstly, 5α-dihydrotestosterone (DHT) binds to AR to promote the association of AR co-regulators. Then the activated AR migrates into nucleus and regulates the expression of target genes in prostate cells (Heinlein and Chang, 2004).

Clinically, PCa is commonly treated by AR pathway perturbation, such as androgen suppression via surgical or chemical castration [gonadotropin-releasing hormone (GnRH) analogs] means (Palmbos and Hussain, 2013). AR antagonist drugs, such as flutamide, nilutamide, bicalutamide, and enzalutamide, take effects by suppressing the action of androgens via competing for AR binding sites (Yamamoto et al., 2012). These androgen blockade therapies are initially effective, however, a considerable population of patients ultimately develop as castration-resistant prostate cancer (CRPC) after prolonged use of an AR antagonist (Schröder, 2008; Yamaoka et al., 2010). AR mutation is one of the leading causes of antiandrogens resistance (Tan et al., 2015). These mutated ARs bind to other steroid hormones and induce the activation of AR transcriptional activity in response to antiandrogens, which results in the PCa growth (Tan et al., 2015). In this case, it shows far-reaching significance to seek and explore novel anti-CRPC drugs targeting gene mutational AR.

DC3 (*Diffusa Cyclotide-3*) is a novel cyclopeptide isolated from the traditional Chinese Medicine (TCM) *Hedyotisdiffusa* (Hu et al., 2015), which has been widely used for the treatment of various cancers and tumors, including prostate cancer, in China with a long history (Lin et al., 2010, 2011; Liu et al., 2010; Lee et al., 2011). It has been experimentally detected that DC3 expresses potent cytotoxicity against LNCaP cells and inhibits the cell migration and invasion. Besides, it can significantly inhibit the development of tumor in weight and size in the mouse xenograft model. All these findings lead to the conclusion that DC3 has evident anti-PCa effects both *in vitro* and *vivo* (Hu et al., 2015). Moreover, in the DC3 sensitivity experiments on three types of human prostate cancer cells, androgen dependent LNCaP cell lines showed obvious higher sensitivity to DC3 comparing to androgen independent PC3 and DUl45 cell lines. Besides, LNCap cell lines typically express T877A-mutated AR, which is the most frequently detected point mutation in CRPC (Veldscholte et al., 1992; Zhou et al., 2010; Yamada et al., 2013). All these evidences suggest that DC3 is a potential candidate binding to T877A AR.

However, the interaction mechanisms between DC3 and T877A-mutated AR are not clear. Therefore, it will be constructive and profoundly significant to launch the mechanism-relevant research. Fortunately, the amino acid sequence of DC3 has been experimentally determined (Hu et al., 2015), which makes it possible to explore the interaction mechanism at the molecular level. Referencing to the published papers, the current research could be consist of three parts: (1) constructing the three-dimensional structure of the cyclopeptide (Jitonnom et al., 2012), in our case is DC3; (2) determining the binding site and binding pose of cyclopeptide to protein (Punkvang et al., 2015); (3) investigating the detailed interaction mechanism between cyclopeptide and protein (Liu et al., 2015; Hitzenberger et al., 2017).

In our study, first of all, homology modeling technology was conducted to construct the three-dimensional structure of cyclopeptide DC3 based on its amino acid sequence. Then molecular docking, all-atom molecular dynamics (MD) simulations and molecular mechanics/generalized Born surface area (MM/GBSA) methods and various MD trajectory analysis methods were combined to explore the most possible binding site of DC3 to AR, investigate the key residues dominant in the binding process, and elucidate the detailed interaction mechanism. The results are expected to reveal the interaction mechanism of DC3-AR complex, promote the development of DC3 and correlative cyclopeptide AR antagonist, which will contribute to the rational drug design for prostate cancer.

## Methods

### Homology modeling of cyclopeptide DC3

Homology modeling is a common technique to construct three-dimensional structure from amino acid sequence using homologous proteins with known structure as templates (Topham et al., 1990; Bordoli et al., 2009; Wang Z. et al., 2015). As amino acid sequence of DC3 was confirmed by Edman degradation and gene cloning (Hu et al., 2015), homology modeling was adopted here to build the 3D structure of DC3 using SWISS-MODEL (Arnold et al., 2006; Guex et al., 2009; Kiefer et al., 2009; Biasini et al., 2014; Bienert et al., 2016). Here, the SWISS-MODEL Template Library (SMTL) is searched both with BLAST and HHblits to identify templates and target-template alignments (Arnold et al., 2006). Then the template was selected based on various criteria such as sequence similarity, sequence identity, coverage, the global quality estimation score (GMQE) and so on.

### Molecular docking analysis of DC3 to AR

Molecular docking (Benkert et al., 2011; Meng et al., 2011; Yuriev et al., 2015) was used to analyze the possible binding site and preferred orientation of DC3 into androgen receptor by simulating combining conformation and computing binding affinity. Here the crystal structure coordinates of the T877A-mutated AR LBD was obtained from the RCSB Protein Data Bank (http://www.rcsb.org/pdb; PDB ID: 4OHA). The missing loop regions were refined by Discovery Studio 2.5. (Accelrys Inc. CA, 2009). Molecular docking process was carried out by using ZDOCK module. The rigid-body protein–protein docking program ZDOCK uses the Fast Fourier Transform algorithm to enable an efficient global docking search on a 3D grid, and utilizes a combination of shape complementarity, electrostatics and statistical potential terms for scoring. Finally, two simple scoring functions–ZRank Score and ZDock Score, pose amount of each cluster, and the rationality of binding mode were taken into consideration to evaluate the docking results.

### Molecular dynamics simulations

Molecular dynamics (MD) simulations were operated through Amber12 package (Case et al., 2012). All the simulations are under the circumstance of ff99SB force field (Hornak et al., 2006) and periodic boundary condition. Firstly, six chloride counterions were added to each system to maintain the electro-neutrality. Then all studied systems were, respectively, immersed into a cubic box of TIP3P (van der Spoel and van Maaren, 2006) water with edge of the box at least 10Å distant from the complex. Energy minimization was carried out in three stages with different harmonic restraint: all atoms constrained by 5.0 kcal·mol^−1^·Å^−2^, only receptor backbone atoms constrained by 3.0 kcal·mol^−1^·Å^−2^ and without any restraint. Each minimization was executed for 5,000 steps, in which the first 2,500 steps were calculated by the steepest descent method while the subsequent 2,500 steps were executed by conjugated gradient method. These systems were heated up to 310.0 K in the NVT ensemble for 100 ps with the receptor backbone atoms constrained by 5.0 kcal·mol^−1^·Å^−2^. And then, a total of 1.5 ns equilibration of each system was performed in NPT ensemble, where the former 800 ps were divided into four stages and the restraints applied to these stages were in a descending order (4.0, 3.0, 2.0, 1.0 kcal·mol^−1^·Å^−2^ respectively), the latter 700 ps were carried out without any restraint. Minimization and heat, as well as equilibration were executed in the Sander program. Finally, a 150 ns production of MD simulations of each system was performed in the PMEMD program at 310.0 K, 1 atm in the NPT ensemble without any restraint. The Langevin thermostat was used to control the temperature and the Berendsen barostat was used for constant pressure simulation. The time step was set as 2fs, and the coordinates of trajectories were recorded every 2 ps. During this simulation, the SHAKE algorithm (Ryckaert et al., 1977) was employed to constrain the bond lengths involving hydrogen, the Particle Mesh Ewald (PME) (Darden et al., 1993; Fischer et al., 2015) was adopted to calculate of electrostatic interaction with a 10Å non-bonded cutoff.

### Free energy calculations

To investigate the interaction of DC3-AR systems from the energetic perspective, the binding free energy calculations based on the trajectories of MD simulations were performed by MM-GBSA method (Hou et al., 2011a,b; Xu et al., 2013; Sun et al., 2014a,b; Chen et al., 2016). The binding free energies ΔG_bind_ was calculated as following equation:

$$\begin{array}{l} \Delta {\text{G}}_{\text{bind}}=\Delta \text{H}-\text{T}\Delta \text{S}=\Delta {\text{E}}_{\text{gas}}+\Delta {\text{G}}_{\text{sol}}-\text{T}\Delta \text{S} \end{array}$$

where ΔH represents enthalpy contribution, which is composed of enthalpy changes in gas-phase (E_gas_) and solvent-phase (ΔG_sol_). –TΔS represents entropy contribution. Entropic calculation is time-consuming, and its value will fluctuate if a small quantity of snapshots were adopted (Hou et al., 2011a; Wang Q. et al., 2015). In this study it was omitted. E_gas_ was considered as the sum of internal interaction (ΔE_int_) from bonds, angles, and torsions, van der Waals (ΔE_vdw_) and electrostatic energies (ΔE_ele_) as follow:

$$\begin{array}{l} \Delta {\text{E}}_{\text{gas}}=\Delta {\text{E}}_{\text{int}}+\Delta {\text{E}}_{\text{ele}}+\Delta {\text{E}}_{\text{vdw}} \end{array}$$

ΔG_sol_ can be decomposed into the polar and nonpolar contributions as follow:

$$\begin{array}{l} \Delta {\text{G}}_{\text{sol}}=\Delta {\text{G}}_{\text{GB}}+\Delta {\text{G}}_{\text{SA}} \end{array}$$

Here, ΔG_GB_ represents the polar solvation contribution, which is calculated by solving GB equation (Kollman et al., 2000; Onufriev et al., 2004). ΔG_SA_, estimated by the solvent accessible surface area, represents the nonpolar solvation contribution.

To further explore the detailed interaction information of DC3-AR complex, free energy decomposition was performed by using MM-GBSA method to identify the key residues responsible for binding energy. The contribution of each residue was calculated without considering the contribution of entropies. The contribution is defined as the sum of van der Waals contribution (ΔE_vdW_), electrostatic contribution (ΔE_ele_), polar solvation contribution (ΔG_GB_), and nonpolar solvation contribution (ΔG_SA_):

$$\begin{array}{l} \Delta {\text{G}}_{\text{residue}}=\Delta {\text{E}}_{\text{vdW}}+\Delta {\text{E}}_{\text{ele}}+\Delta {\text{G}}_{\text{GB}}+\Delta {\text{G}}_{\text{SA}} \end{array}$$

Snapshots, used for both binding free energy and free energy decomposition calculations, were extracted from the last 50 ns of MD trajectories at intervals of 2 ps.

### MD trajectory analysis

#### Hydrogen bond analysis

The number of formed hydrogen bonds vs. simulation time was calculated to detect the system stability during the process of simulation. Here, the hydrogen bond criteria was set as the distance of acceptor-donor <0.35 nm and the angle >120° (Fu et al., 2013). 0.35 nm is a common choice of hydrogen bond distance in literature (Liu et al., 2014; Wang Q. et al., 2015). The frames adopted for this calculation were extracted from the whole 150 ns MD trajectories at intervals of 2 ps. Besides, in order to determine exactly how hydrogen bonds play dominant roles in maintaining system stability in the last 50 ns MD simulations, the occupations of hydrogen bonds formed in this period were calculated as following equation (Liu et al., 2014):

$$\begin{array}{l} P_{hbond}=\frac{N_{exist}}{N_{total}}\times 100\% \end{array}$$

Where N_exist_ is the number of frames which formed targeted hydrogen bond, and N_total_ is the total number of frames. The occupations are varied from 1 to 100%, and a higher percentage represents a more stable-existed hydrogen bond.

#### Dynamic cross correlation matrix

The dynamic cross-correlation matrix (DCCM) analysis of the Cα atoms during the last 50 ns of the first parallel trajectory was performed to explore the correlated motion between residues of DC3-AR complex. The cross correlation matrix C_ij_, which reflects the displacements of the Cα atoms relative to average positions, was determined by following equation (Lange et al., 2005; Ghosh and Vishveshwara, 2007):

$$\begin{array}{l} C_{ij}=\frac{\langle \Delta R_{i\;}\cdot \Delta R_{j}\rangle }{\sqrt{\langle \Delta R_{i\;}\cdot \Delta R_{i\;}\rangle \langle \Delta R_{j}\cdot \Delta R_{j}\rangle }} \end{array}$$

Where ΔR_*i*_ and ΔR_*j*_ represent displacements of atom *i* and *j*, respectively. The value of C_*ij*_ fluctuated from −1 to 1, the positive value indicates a correlated motion between the residue *i* and residue *j*, while negative values indicates an anti-correlated motion.

#### Clustering analysis

Clustering analysis was conducted by using the MMTSB toolset in Amber 12 to determine the representative structure of DC3-AR complex during the last 50 ns MD simulations. Firstly, the similar conformations of DC3-AR complex generated from the trajectory were classified into one cluster, and the most populated cluster has maximum number of conformations. Centroids of the generated clusters were then calculated and generated. Subsequently, RMSD of each structure was calculated with respect to specific centroid. Ultimately, the structure with lowest RMSD to cluster centroid from the most populated cluster was defined as the representative structure of DC3-AR. After that, the representative structure was adopted to generate the hydrophobic and electrostatic interaction surface of DC3-AR complex by using UCSF Chimera package (Pettersen et al., 2004; http://www.cgl.ucsf.edu/chimera).

#### Dynamical correlation network

The cross-correlation matrix C_*ij*_ was also employed to build the dynamical correlation network to intuitively exhibit the correlated motion between residues in different protein domains. The Cα atom of each residue was defined as a “node,” and “edge” is the connection of each pair of nodes if the residue pairs interact with each other (Liu et al., 2014). The edges were computed as the following equation:

$$\begin{array}{l} d_{ij}=-\log\left(\left|C_{ij}\right|\right) \end{array}$$

Here, each edge has a specific contribution to the movement of complex, the motion of residue *i* can be used to predict the motion direction of residue *j*. If |*i* − *j*|<=10, cross-correlation between *i* and *j* are ignored to remove the correlations due to special closeness. Besides, −0.3 ≤ *C*_*ij*_ ≤ 0.3 were also deleted to make network plot more concise. Network View plugin in visual Molecular Dynamics 1.9.2 (VMD) (Humphrey et al., 1996; Hsin et al., 2008) was used to visualize the interaction network.

## Results and discussion

### Homology modeling of cyclopeptide DC3

**Figure 1:**
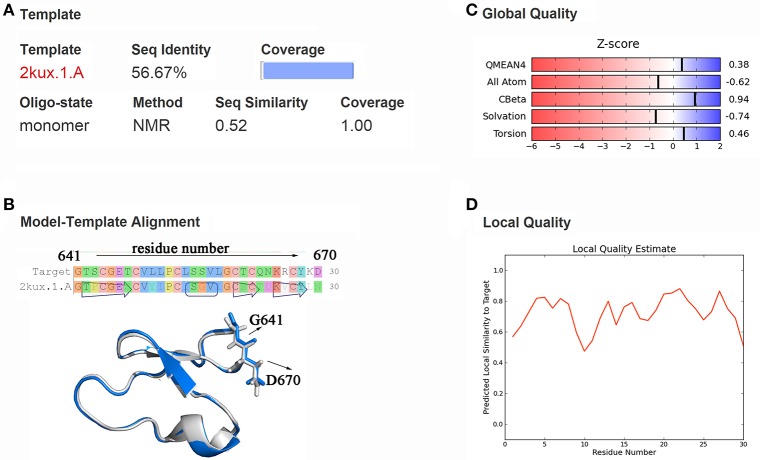
Homology modeling results. **(A)** Information about template, sequence identity, sequence similarity, coverage and GMQE, QMEAN values. **(B)** Sequence and structure alignment of target and template. The constructed DC3 structure is shown in marine ribbon and the template structure in gray ribbon. G641 and D670 are shown in sticks to exhibit the cyclization state of DC3. **(C)** Z-score information of the constructed model. **(D)** The predicted local similarity of each residue to target.

Through templates searching by SWISS-MODEL, 28 qualified templates for DC3 sequence were found. According to the selection criteria and the basic structure characters of cyclopeptide (Craik et al., 1999; Sze et al., 2009) 2kux.1.A (Plan et al., 2010) was selected as the final template to construct the three-dimensional structure of DC3. As shown in Figure 1A, the amino acid sequence identity between template and target is 56.67%, sequence similarity is 0.52, Global Model Quality Estimation (GMQE) value is 0.94, and the target sequence is all covered. The sequence alignment and structure comparison of target and template were shown in Figure 1B, from where highly similarity can be easily observed. Besides, the Z-score information and predicted local similarity of each residue to target were shown in Figures 1C,D, respectively. All these information reflects the reliability of the constructed model. As the AR residues were numbered from 671 to 919, here, the number of DC3-residues was defined from 641 to 670 for convenience.

### Binding site exploration

#### Molecular docking analysis

**Figure 2:**
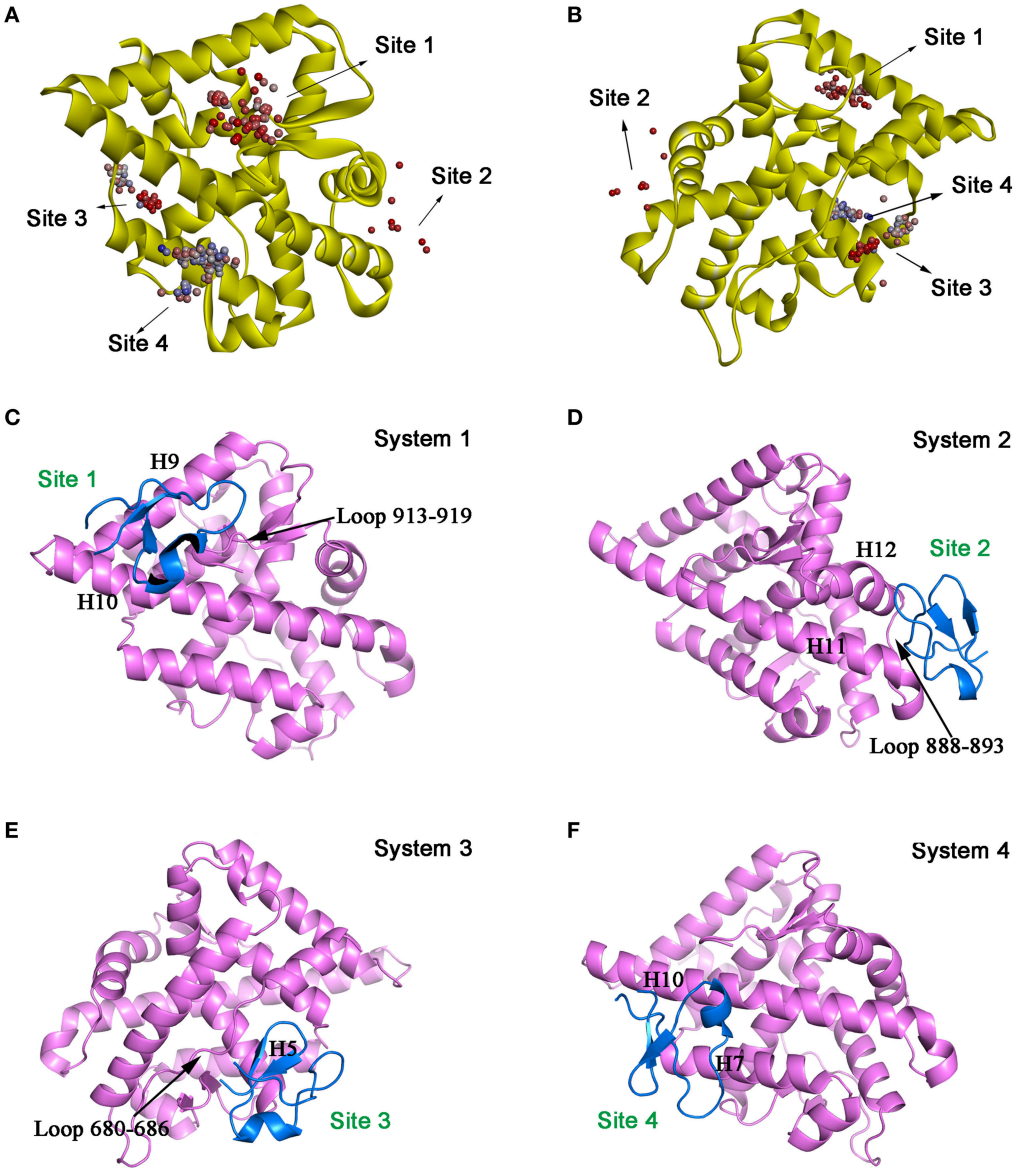
The top four possible binding modes of DC3-AR complex. **(A,B)** AR is shown in yellow ribbon, and each dot represent a DC3 conformation. The color of dot from red to blue indicates the ZRank Score from high to low. **(C–F)** Four initial structures of DC3-AR complex selected form the top four possible binding sites for MD simulations. DC3 is shown in marine and AR is shown in violet.

Molecular docking was then performed to explore the possible binding site and binding mechanism of DC3-AR complex. As a result, 2,000 poses of 60 clusters were generated by ZDOCK module. The pose amount of each cluster, ZRank Score, ZDock Score, and the rationality of binding mode were combined to assess the poses. The ZRank Score represents the extent of energy contribution to the system when a ligand binds to a receptor. The ZDock Score is calculated based on the shape matching degree of receptor and ligand, and a higher score represents a better pose. Furthermore, the electrostatic interaction, Van der Waals' force, and desolvation energy were also taken into consideration. A lower score represents a better pose. Based on these criteria, top four possible binding sites of DC3 to AR were selected. As shown in Figures 2A,B, the top four clusters of DC3-ARs located in Site 1, Site 2, Site 4, and Site 3, respectively. The score of binding to Site 2 was higher than other possible binding sites although the pose number of this cluster is relatively small. However, it should be noted that scoring functions do not always yield the best predictions of binding affinity (Ramírez and Caballero, 2016). To further confirm the binding affinity prediction, Molecular dynamics (MD) simulations were subsequently performed to obtain more conformational sampling of these four systems. Four poses (Table 1, Figures 2C–F) of DC3-AR complex were determined as the initial structures, named as system 1,system 2, system 3, and system 4, to perform MD simulations, which were selected from the top four possible binding sites with good ZRank Score, fine ZDock Score, and rational conformations.

**Table 1:** The selected top four possible binding sites of DC3-AR complex and the corresponding poses scoring.

**System**	**ZRank score (kcal/mol)**	**ZDock score**	**Binding site**
1	−85.450	11.66	Site1: Helix 9, Helix 10, Loop 913-919
2	−97.309	10.92	Site2: Helix 11, Loop 888-893, Helix 12
3	−92.708	13.08	Site3: Loop 680-686,Helix 5
4	−78.121	12.00	Site4: Helix 7, Helix 10

#### Root mean square deviation

**Figure 3:**
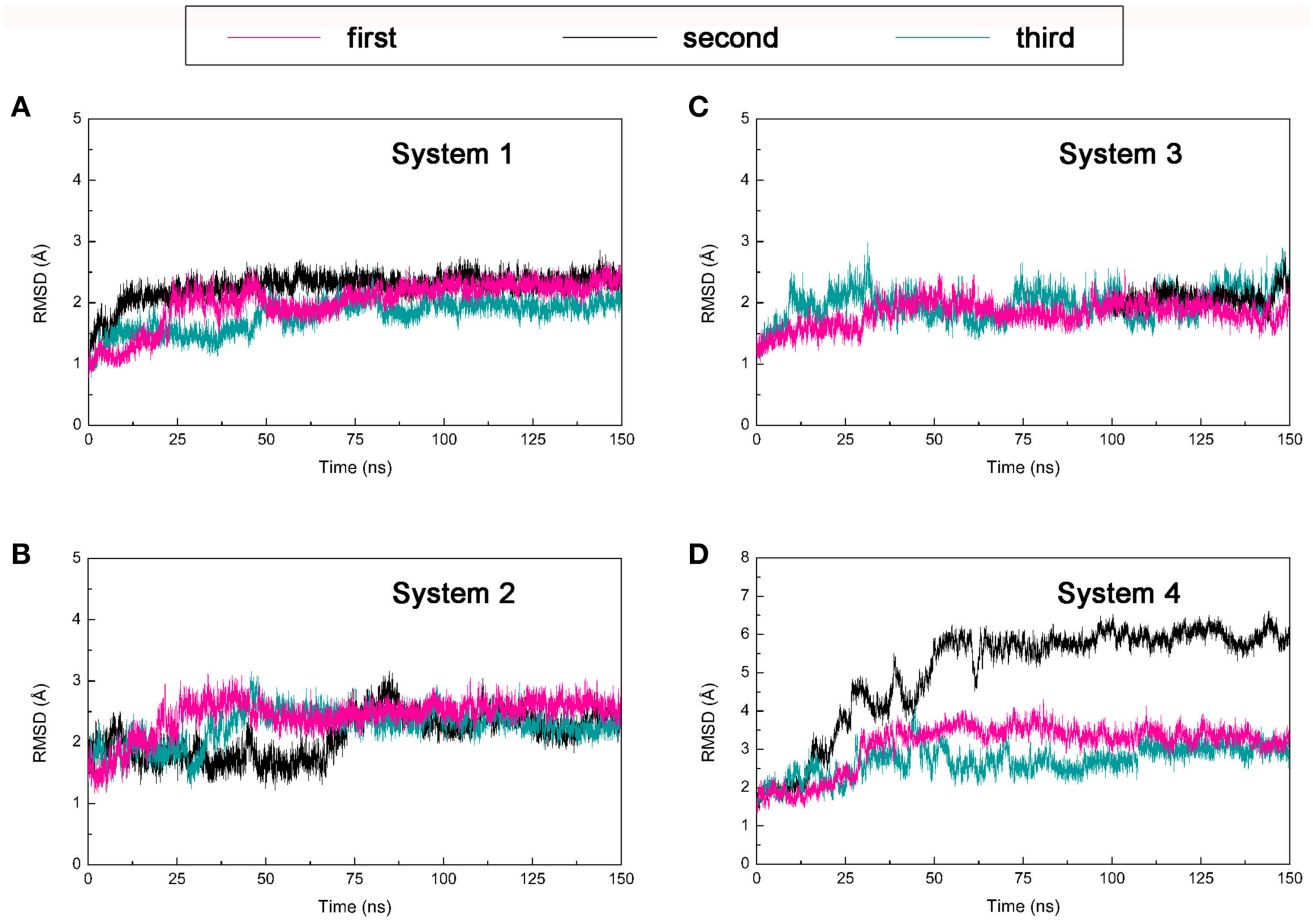
Root mean square deviation (RMSD) of backbone atoms of four DC3-AR complexes systems from three parallel trajectories using the initial structure as reference. **(A)** RMSD of system 1. **(B)** RMSD of system 2. **(C)** RMSD of system 3. **(D)** RMSD of system 4.

One hundred and fifty nano seconds MD simulations were calculated on the four DC3-AR complexes systems acquired from molecular docking were then performed respectively. To obtain reliable and repeatable results, three parallel MD simulations processes were executed on each system. Then root man square deviation (RMSD) values of DC3-AR complexes backbone atoms were calculated relative to the initial structures to monitor the stability and overall convergence of each system during the simulation process. As shown in Figure 3, all systems experienced various degrees of fluctuations at first, but gradually tended to converge. It can be seen that, the first and third trajectories of system 4 and all parallel trajectories of other three systems reached equilibrium in the last 50 ns, which were qualified for subsequent analyses of the dynamic behavior. However, the second parallel trajectory of system 4 experienced great structure changes at about 50 ns, which suggested the relatively poor stability of system 4. Considering the abnormality, this trajectory was eliminated in the succeeding binding free energy analysis which was carried out by averaging the values of parallel trajectories.

#### Root mean square fluctuation

**Figure 4:**
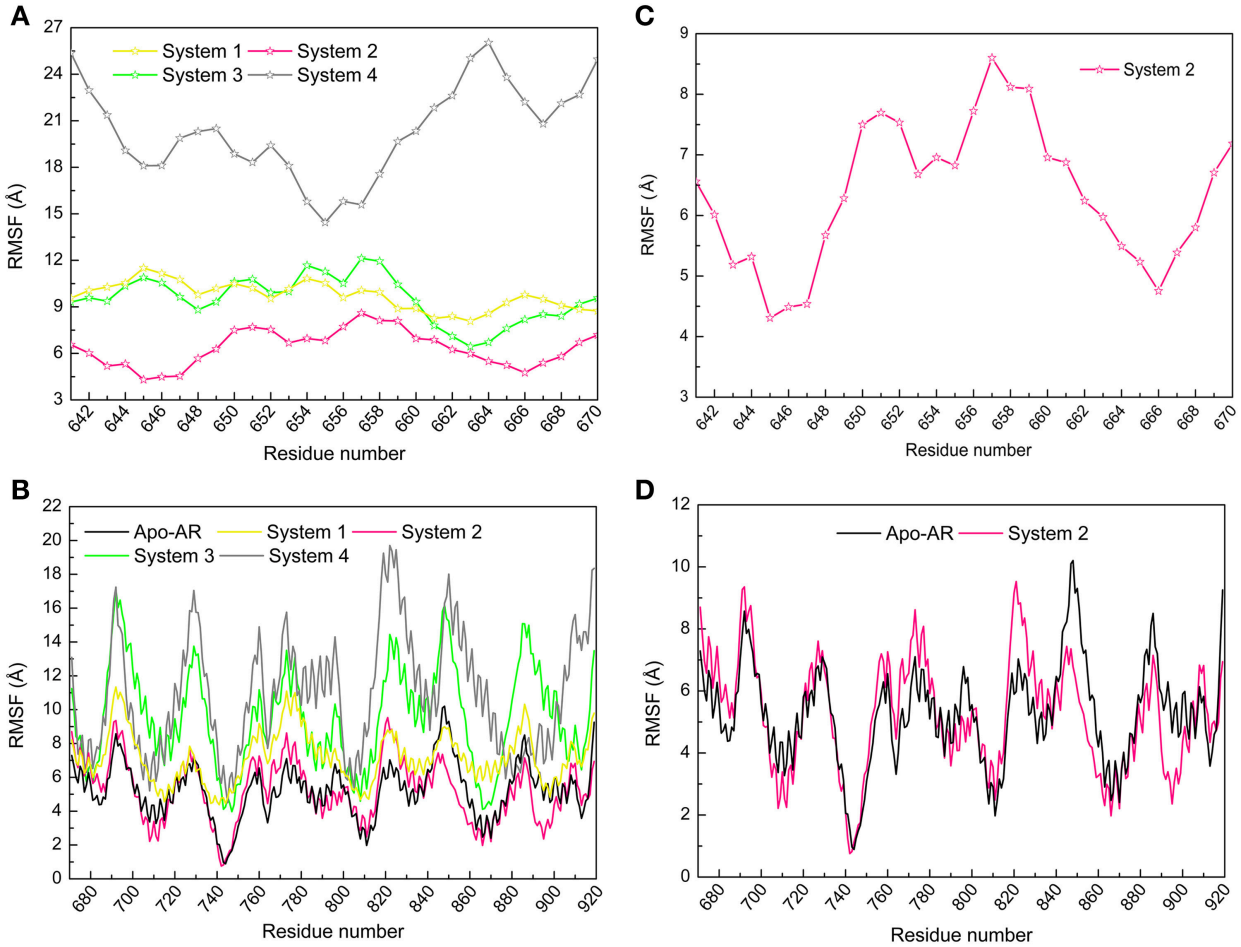
Root mean square fluctuation (RMSF) values of Cα atoms calculated by the first parallel trajectory of studied systems in the last 50 ns MD simulation. **(A)** RMSF values of DC3 residues in four systems. **(B)** RMSF values of DC3 residues in system2. **(C)** RMSF values of AR residues in four systems and apo-AR system. **(D)** RMSF values of AR residues in system 2 and apo-AR system.

The root mean square fluctuation (RMSF) reveals the fluctuation of certain residues during simulation process around its average position, which is also a tool to assess the dynamics stability of system. Here, RMSF values of Cα atoms in the last 50 ns were calculated by employing the first parallel trajectory of each system. RMSF of DC3 residues were shown in Figure 4A to explore the stability of the DC3. It can be seen that, different from the great fluctuation in other systems, DC3 residues in system 2 experienced minor motions. It demonstrated that DC3 in system 2 showed obvious superiority in the stability. RMSF of AR residues in these four systems were compared with apo-AR system (made up by AR only) to determine whether the binding of DC3 affects the stability of AR. As shown in Figure 4B, the overall RMSF of system 2 is lower than apo-AR system, especially the residues 840-870 (corresponding to H9, loop 843-849, H10), 880-905 (corresponding to H10, H11, loop888-893, H12). Moreover, only in apo-AR system and system 2, the RMSF values of all residues were under 10Å. Whereas, residues in other systems showed apparently larger conformational changes comparing to apo-AR system. These results demonstrated that the combination of DC3 to AR in site 2 (corresponding to Helix 11, loop 888-893, Helix 12) could stabilize androgen receptor. However, DC3 combination in other sites could visibly reduce AR stability. These RMSF analyses indicated that site 2 is the most possible site of DC3 binding to AR.

#### Interaction energetic features

In order to explore the interaction energetic features of DC3-AR complexes, MM-GBSA method was employed to calculate the binding free energies of each system. The average binding free energies and detailed energetic contribution components of the last 50 ns of parallel trajectories were calculated and shown in Table 2. It can be seen that the free energy of system 2 (−40.94 kcal/mol) is apparently lower than system 1 (−33.49 kcal/mol), system 3 (−18.99 kcal/mol), and system 4 (−19.73 kcal/mol). It demonstrated that DC3 showed a higher binding affinity to AR in system 2 comparing to other systems, which indicated that DC3 has a great tendency to bind to AR in site 2 and system 2 is more likely to remain stable. This result conforms to the conclusion obtained from the previous RMSF analysis. Moreover, details of the dominant components driving DC3 to bind to AR can be acquired by dissecting the binding free energy into contributing components. Here, the electrostatic interaction (ΔE_ele_) in system 2 (−232.64 kcal/mol) can be found to make a great contribution to the low binding free energy of the whole system, which reflects that significant electrostatic interactions may exist between DC3-AR complex and contribute greatly to the system stability.

**Table 2:** Binding free energy and the detailed energetic contribution components of four systems of DC3-AR complex averaged by the last 50 ns of parallel trajectories (kcal/mol).

**Contributions**	**System 1**	**System 2**	**System 3**	**System 4**
E_vdW_	−70.98(2.97)	−50.84(0.06)	−44.89(1.50)	−44.18(4.74)
E_ele_	−22.58(19.09)	−232.64(25.84)	−13.28(8.82)	−36.53(10.14)
G_GB_	69.34(18.17)	250.09(26.80)	44.79(7.60)	67.15(6.13)
G_SA_	−9.27(0.53)	−7.55(0.23)	−5.61(0.28)	−6.16(0.82)
E_gas_	−93.56(18.46)	−283.48(25.78)	−58.17(8.69)	−80.71(5.40)
E_solv_	60.06(17.70)	242.54(26.57)	39.18(7.74)	60.99(6.95)
ΔG_bind_	−33.49(7.10)	−40.94(0.79)	−18.99(2.18)	−19.73(1.56)

#### Dynamic cross-correlation matrices analysis

**Figure 5:**
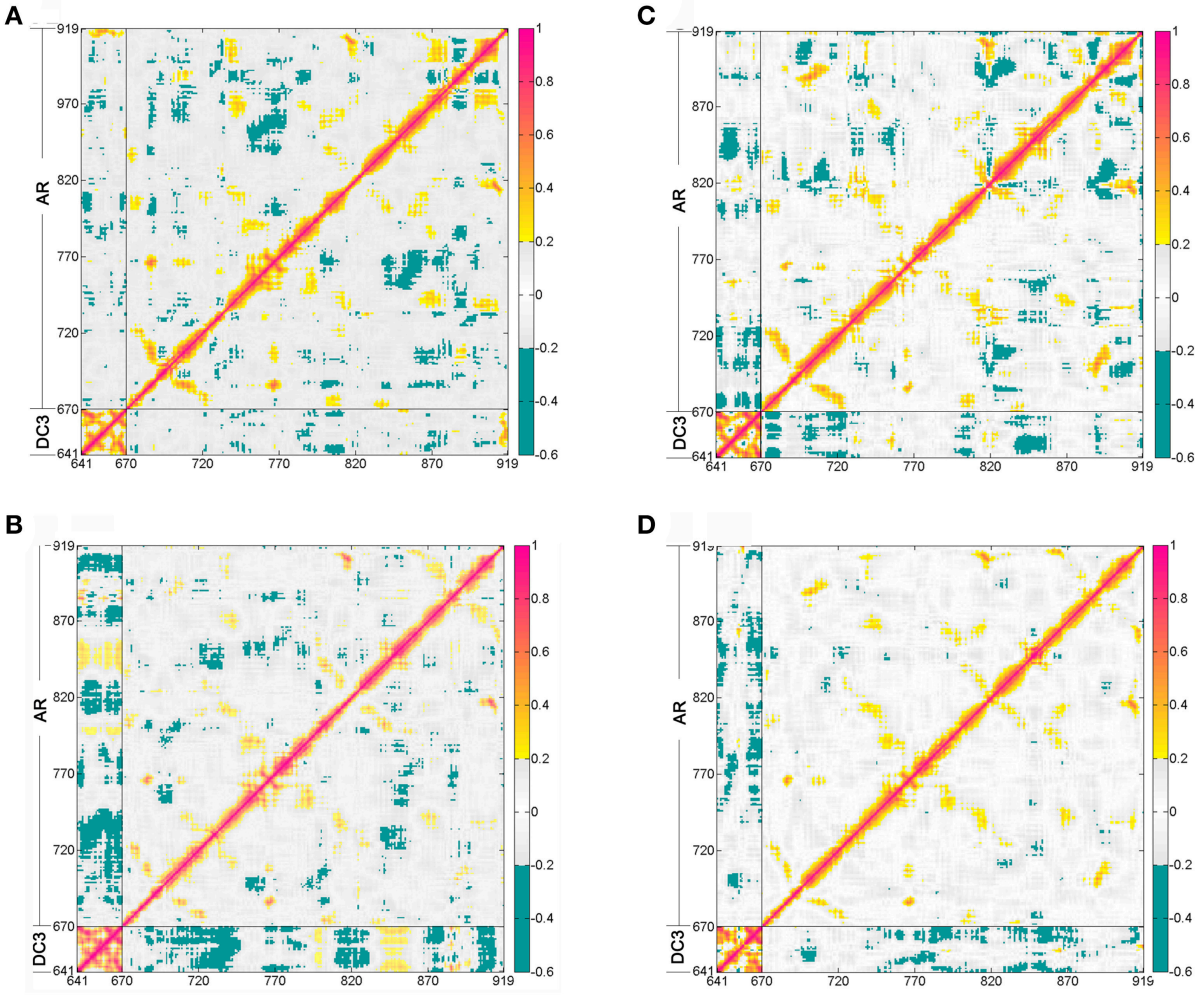
Dynamic cross-correlation matrices (DCCM) of the Cα atoms around their mean positions during the last 50 ns trajectory from the first parallel trajectory. The degrees of correlation and anti-correlation correspond to the color bar. **(A)** System 1. **(B)** System 2. **(C)** System 3. **(D)** System 4.

The dynamic cross-correlation matrices (DCCM) analysis was further analyzed (Figure 5) to investigate the correlated conformational motions of DC3-AR complexes. Here, highly positive regions (colored by red and yellow) are associated with strong correlated motions (residue pairs move in the same direction), while negative regions (colored by blue) are linked with strong anti-correlation movements (residue pairs move in the opposite direction). Inspecting the DC3 domains of the four systems, it can be observed that relatively stronger correlations exist between DC3 residues in system 2. Moreover, comparing to other systems, obviously more correlated and anti-correlated motions between DC3 residues and AR residues can be found in system 2. These obvious differences indicated that there were more and stronger cross-correlation motions between residues in system 2, demonstrating more intense interaction and better stability of this DC3-AR complex.

#### Hydrogen bonds analysis

**Figure 6:**
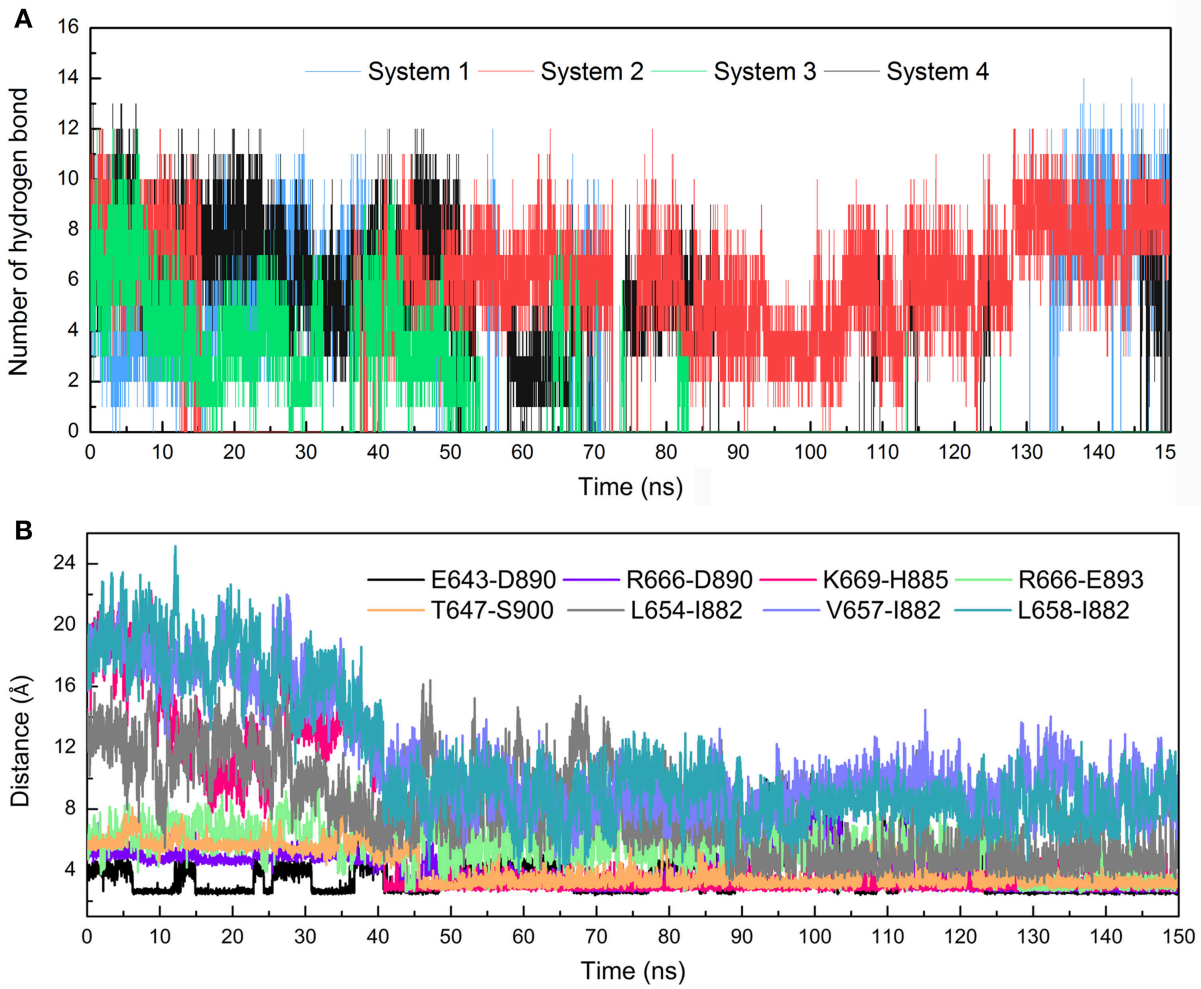
Hydrogen bonds and Distance analyses results. **(A)** Number of hydrogen bonds formed between DC3 and AR vs. simulation time calculated from the first parallel MD simulation of studied systems. **(B)** Distance of key residue pairs vs. simulation time calculated from the first parallel MD simulation of system 2.

The stronger cross-correlation between DC3 and AR residues found in system 2 might also due to the formation of hydrogen bonds during MD simulations. Hydrogen bonds, as critical indicators of nonbonding interactions, play vital roles in the protein-ligand recognition process (Ramírez and Caballero, 2016). During this MD simulation, the number of hydrogen bonds formed between DC3 and AR vs. simulation time was calculated and plotted in Figure 6. Though we set 0.35 nm as the hydrogen bond criteria in this study, the distance we calculated for hydrogen bond were almost all around 3 Angstrom, long-distance hydrogen bond do not exist. As shown in this figure, hydrogen bond interaction patterns formed in system 2 remained constant during the entire simulation time. While in other systems, hydrogen bonds were unstable and most of them disappeared in about 90 ns. Even in the last 50 ns of simulation, the amount of hydrogen bonds still fluctuated a lot. This result reflects the obvious stability of system 2, which further proves that DC3 tends to bind to AR at site 2 as previous RMSF and binding free energy analyses demonstrated. Besides, the intermolecular hydrogen bonds formed in the last 50 ns MD simulations with occupation more than 10% were listed in Table 3. It can be clearly observed that much more hydrogen bonds are stably formed in system 2. On one hand, there are 13 hydrogen bonds occupied more than 10% in system 2, while only 4 hydrogen bonds in system 1, one hydrogen bond in system 3, and even no one in system 4. On the other hand, the highest occupation of hydrogen bonds are 23.51 and 12.55% in system 1 and system 3, respectively, while in system 2 hydrogen bonds formed between K669-H885, D890-R666, S900-T647, and D890-E643 occupied 85.23, 74.54, 72.49, and 64.38%, respectively.

**Table 3:** The hydrogen bonds formed between DC3 and AR with occupation more than 10% in the last 50 ns MD simulations of four systems.

**Complex**	**Acceptor**	**Donor**	**Frames**	**Frac**	**AvgDist**	**AvgAng**
System 1	F916@O	C662@N	5878	0.2351	2.8803	161.0701
	S853@OG	D670@N	4556	0.1822	3.0546	153.5131
	S656@OG	T918@N	3965	0.1586	3.1798	154.4460
	C653@O	T918@OG1	3145	0.1258	2.7764	161.6225
System 2	K669@O	H885@NE2	20880	0.8352	2.9836	148.9214
	D890@OD1	R666@NH2	18635	0.7454	2.8981	155.1367
	S900@OG	T647@N	18123	0.7249	3.1593	156.3738
	D890@OD2	R666@NH1	16246	0.6498	2.8350	159.6511
	D890@OD2	S643@OG	16094	0.6438	2.6662	164.7336
	D890@OD2	R666@NH2	15971	0.6388	3.1114	140.8035
	E893@OE2	R666@NE	8377	0.3351	3.0023	148.8850
	E893@OE1	R666@NE	8179	0.3272	3.0252	148.1640
	D890@OD1	S643@OG	6708	0.2683	2.7365	161.1178
	E893@OE1	R666@NH2	5978	0.2391	2.8769	153.9622
	F891@O	R666@NH2	5776	0.2310	2.8978	140.2469
	E893@OE2	R666@NH2	5165	0.2066	2.8858	153.5793
	D890@OD1	R666@NH1	3571	0.1428	3.2415	137.6629
System 3	T755@O	C653@N	3137	0.1255	2.9959	158.6020
System 4	–	–	–	–	–	–

Based on these data, it can be concluded that site 2 (H11, loop888-893, H12) is the most possible site of DC3 binding to AR complex.

### Exploration of the binding mechanism between DC3 and AR site 2

To fully explore the binding modes and interaction mechanisms of DC3 and AR, system 2 was further studied to reveal the complicated binding mechanism of DC3-AR complex.

#### Root mean square fluctuation analysis

According to the RMSF values of DC3 residues and AR residues shown in Figures 4C,D, respectively, it is easy to determine the key residues dominant in the binding process of DC3-AR complex. In DC3, residues 645-647 and 666 with low RMSF values experienced minor fluctuation, which indicated these residues were relatively more stable during the simulation process. Comparing the AR residues in system 2 to apo-AR system, it can be observed that the RMSF values of residues 840-870 (correspond to H9, loop 843-849, H10), 790-800 (correspond to H7, loop 797-800) in system 2 were lower than apo-AR. which revealed the definite role of these residues in maintaining the system stability. Moreover, residues of binding site 2 (residues 880-903, correspond to H11, loop 888-893, H12) also exhibited obvious lower fluctuation comparing to apo-AR system. It can be observed that the binding site has become one of the most stable regions in system 2. These results not only indicated the dominant role of these residues, but also suggested that specific interactions must have been formed between residues 880-903 and DC3 residues, which then constrained the mobility of them and made the whole system stable.

#### Clustering analysis

**Figure 7:**
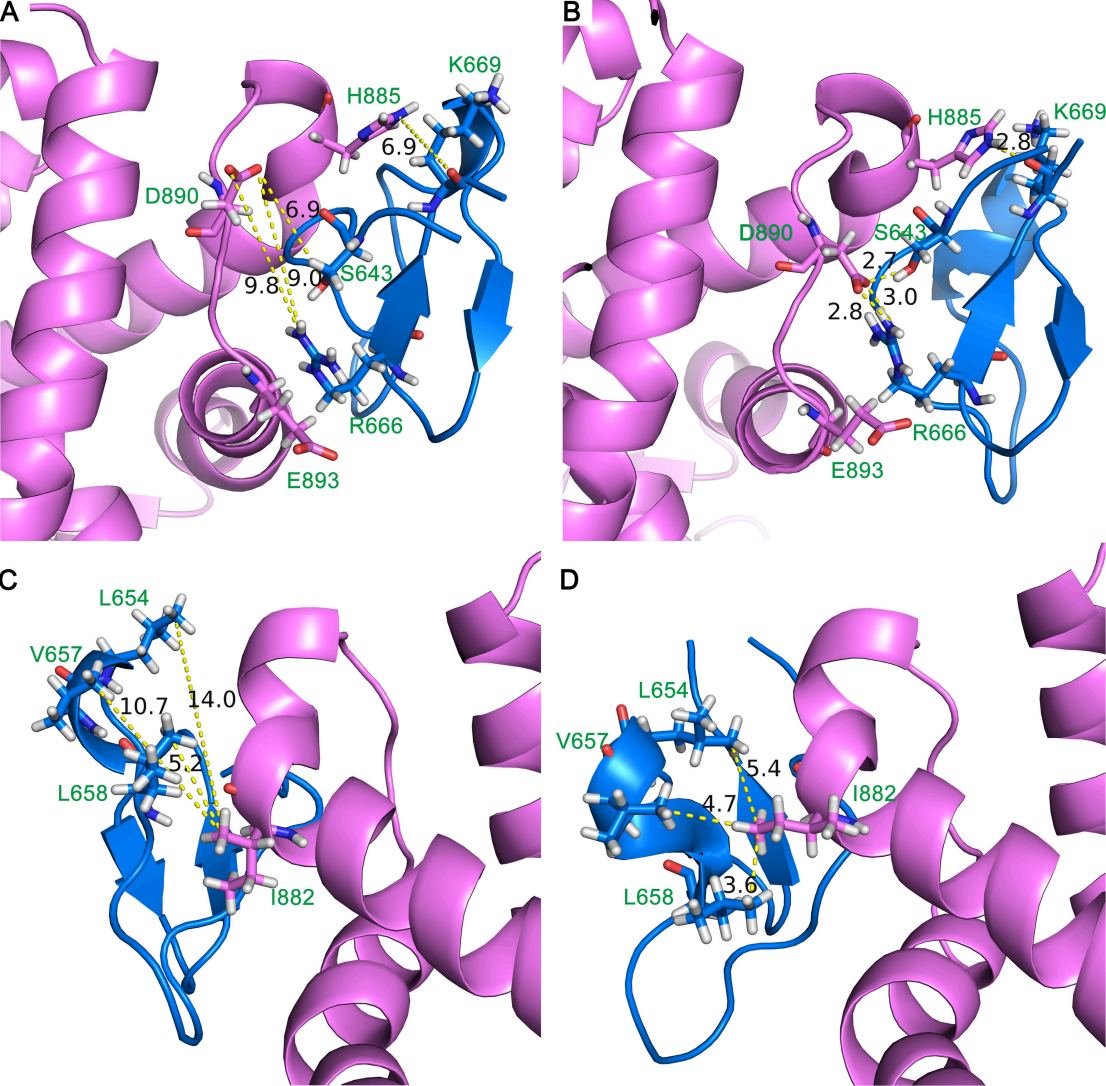
Initial structure **(A,C)** and representative structure **(B,D)** of the binding site of DC3-AR complex. DC3 is shown in marine and AR is shown in violet. Key residues are shown in stick, and yellow dashed lines represent the distance of specific residue pairs.

The representative conformation of DC3-AR complex during MD simulation was extracted by clustering analysis. The first parallel trajectory of system 2 was grouped into 4 clusters based on the conformational similarity. The most populated cluster contained14068 frames, which accounted for 56.27% of all frames extracted from the last 50 ns MD simulation. Then the conformation with least RMSD value in most populated cluster was defined as the representative structure. Here, the representative structure of DC3-AR complex extracted from MD simulations trajectory and the initial structure acquired from molecular docking were plotted in Figures 7A,B, respectively, to exhibit the interaction of key residues visually. From these two figures, it can be seen that residues pairs K669-H885, R666-D890, S643-D890, and R666-E893 were apparently drawn near to each other during the MD simulations. All these indicated that some significant interaction forces might formed between these residues, which further stabilized the complex as RMSF data verified. Similarly, the closeness of residues L654-I882, V657-I882, L658-I882 could also be easily observed in MD simulation through Figures 7C,D. As residues L654, V657, L658, and I882 are nonpolar amino acid, it suggests specific hydrophobic interactions may formed, which needs to be further validated.

#### Interaction surface exploration and free energy decomposition

**Figure 8:**
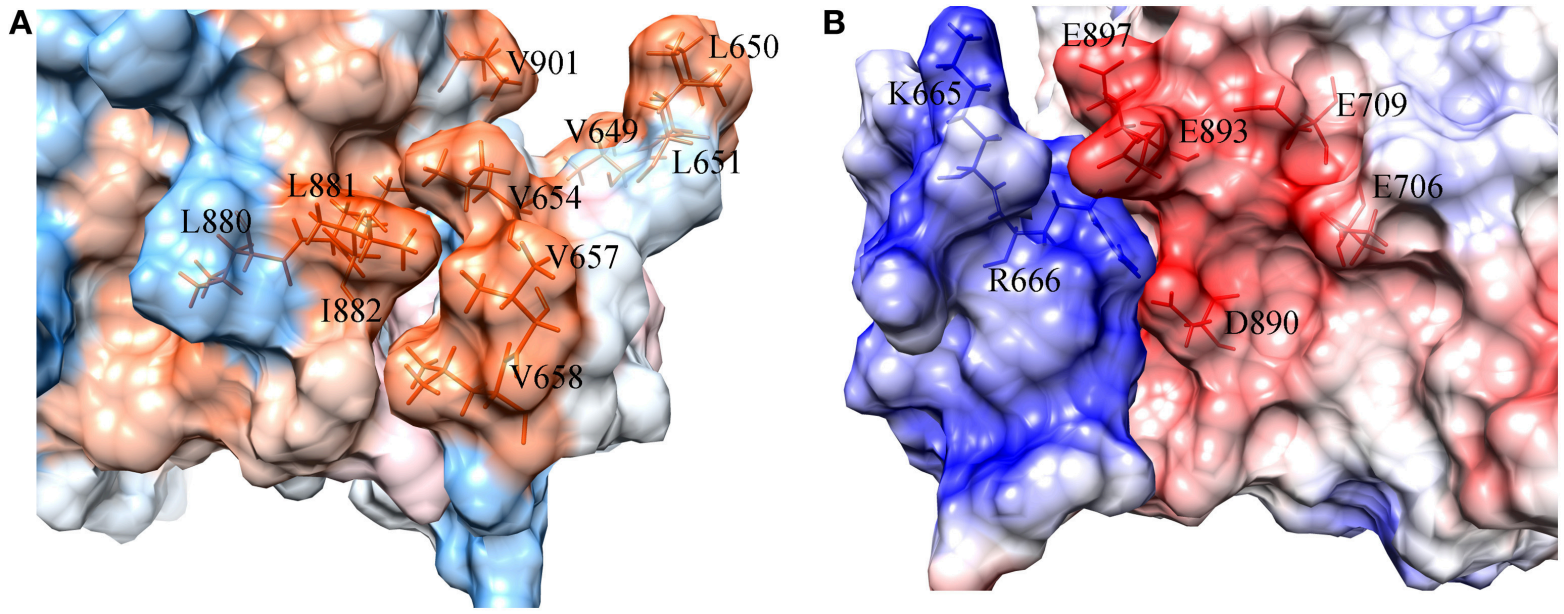
Surface of hydrophobic interaction **(A)** and electrostatic interaction **(B)** in the binding site of DC3-AR complex. **(A)** Dodger blue represents hydrophobic minimum, orange represents hydrophobic maximum, key residues are indicated as orange sticks. **(B)** Positively charged domain colored by blue, negatively charged domain colored by red, key residues are indicated as blue and red sticks.

To figure out the binding mechanism between the key residues in the binding process of DC3-AR complex, hydrophobic and electrostatic interaction surfaces of representative structure were generated. The hydrophobic interaction surface was plotted in Figure 8A, where dodger blue represents hydrophobic minimum, gray depicts the hydrophobicity of 0, and orange represents the largest hydrophobicity. It can be observed that the binding site of AR indeed exits strong hydrophobic interactions with DC3, which promoted the identification and combination of ligand and receptor to a certain extent. Furthermore, two highly hydrophobic interaction domains (deep orange) formed by V649, L650, L651-V901, and L654, V657, L658-L880, L881, I882, respectively, can be found, which played dominant roles in the development of hydrophobic interaction.

**Figure 9:**
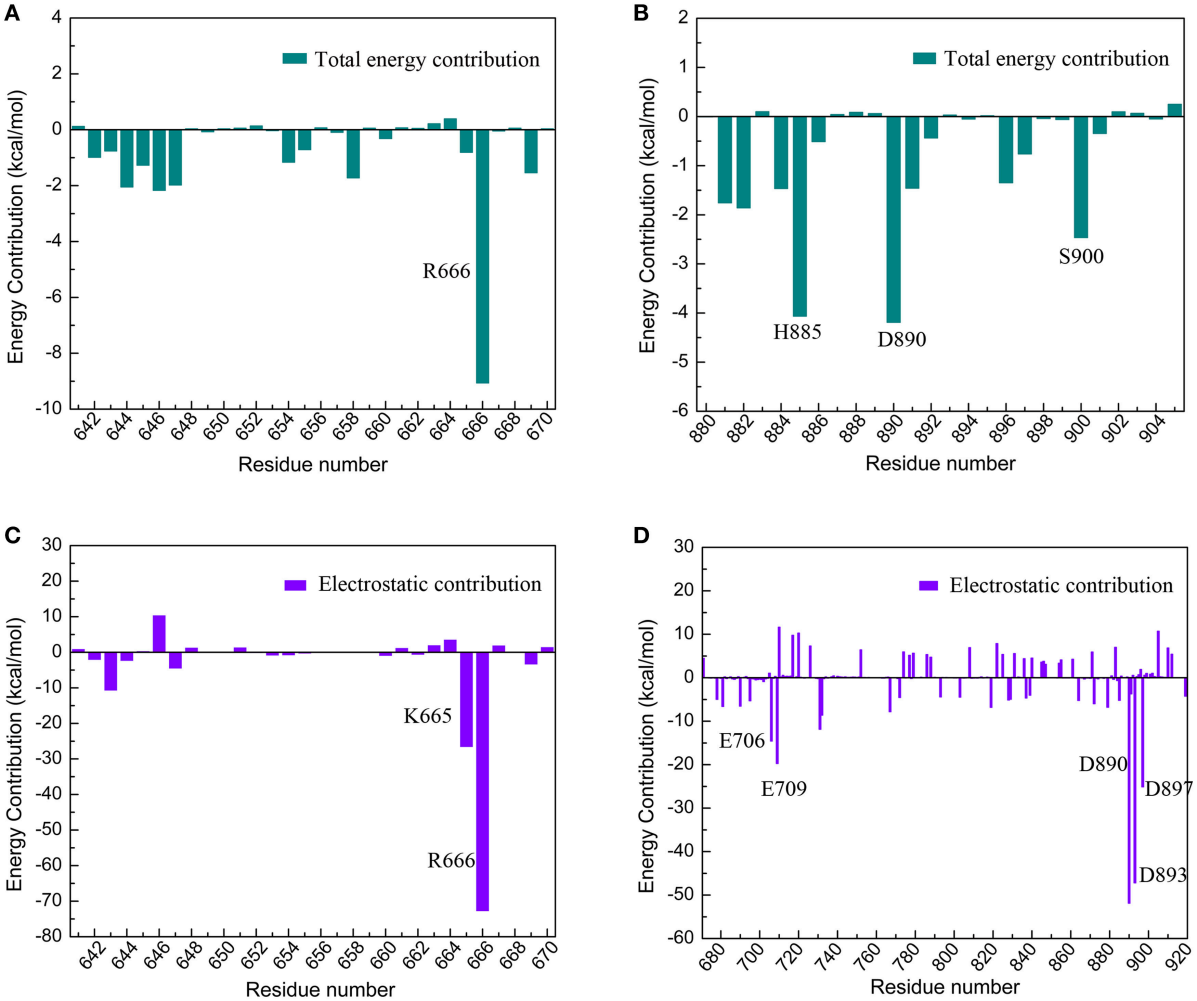
Free-energy decomposition of DC3-AR complex of the first parallel MD simulation in the last 50 ns MD simulation. **(A)** Energy contribution of DC3 residues. **(B)** Energy contribution of AR residues in the binding site. **(C)** Electrostatic contribution of DC3 residues. **(D)** Electrostatic contribution of AR residues.

The electrostatic interaction surface was shown in Figure 8B. It can be seen that most DC3 residues carry positive charge, which come into being a positively charged surface (blue) in the interface. Whereas, a certain number of negatively charged residues (red) existed in binding site of AR. These residues with opposite charges in the interface attracted each other, and made great contribution to the binding process. To deeply investigate the energetic contribution, especially the electrostatic contribution of key residues, free-energy decomposition was performed based on the last 50 ns MD simulations of system 2. The energy contributions of DC3 and AR residues were shown in Figures 9A,B. The electrostatic contributions of DC3 and AR residues were depicted in Figures 9C,D respectively. From the energy contribution it can be seen that residue R666 made incomparable contribution to free-energy, which demonstrated that interactions existed between R666 and AR residues played essential roles in DC3-AR binding process. Meanwhile residues H885, D890, and S900 dominated the energetic contribution. These results proved that residues of both DC3 and AR in binding site do make contributions in the decrease of free energy. Combined with the hydrogen bond analyzed before, it comes to a conclusion that hydrogen bonds formed between K669-H885, D890-R666, S900-T647, and D890-E643 are especially critical components to the interaction between DC3 and AR. Based on all the results above, we can reach a conclusion that K665, R666 of DC3 and E706, E709, D890, E893, E897 of AR ultimately make great contributions to the binding process.

#### Distance analysis

To further validate the interaction formed between key residues and investigate the formation process, the distances of key residue pairs mentioned above vs. simulation time were calculated and plotted in Figure 6B. It can be firstly observed that the distance of residue pairs K669-H885, L654-I882, V657-I882, and L658-I882 experienced an obvious decrease in about 40 ns and maintained stable in the later simulation. Significant conformational changes of binding site could be speculated based on this crucial distance variation. This result revalidated the formation of hydrogen bond between K669-H885, and hydrophobic interaction between L645-I882, V657-I882, L658-I882. In addition, residue pairs E643-D890, T647-S900, R666-D890, and R666-E893 kept highly close (about 5Å) and remain stable throughout the simulation process, and some of them even reached about 2.5Å in the last 50 ns. Besides, from the hydrogen bonds figure shown in Figure 6A, it can also be seen that certain hydrogen bonds formed in about 40 ns. All these results proved the stable existence of hydrogen bonds and electrostatic interaction during the whole MD simulations.

#### Cross-correlation networks

**Figure 10:**
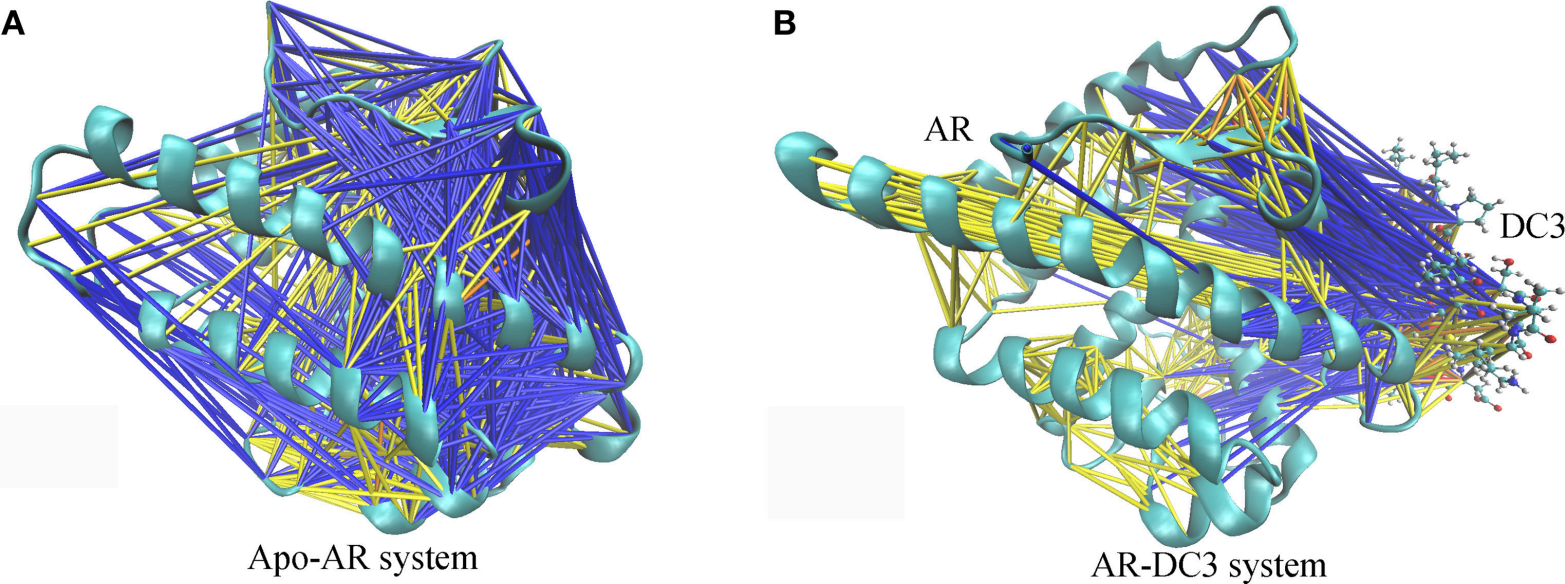
Cross-correlation networks of apo-AR system **(A)** and DC3-AR system **(B)**. AR is shown in cyan cartoon and DC3 is shown as CPK model. Cross-correlation of residue pairs are distinguished by colors. Correlation are indicated by yellow (0.3 ≤ *C*_*ij*_ < 0.5), orange (0.5 ≤ *C*_*ij*_ < 0.7) and red (*C*_*ij*_ ≥ 0.7). Anti-correlation are indicated by blue (−0.5 < *C*_*ij*_ ≤ −0.3) and black (−0.7 < *C*_*ij*_ ≤ −0.5).

To characterize and intuitively exhibit the underlying dynamical cross-correlations among different parts of DC3-AR complex, the overall cross-correlation networks of system 2 and apo-AR system were constructed. As shown in Figure 10A, masses of correlation and anti-correlation both widely and simultaneously existed in apo-AR system, which made it hard to identify the specific cross-correlation pattern, in other words, the interaction between different protein domains was mixed and disorderly. However, when DC3 binding to AR (Figure 10B), all anti-correlation among different AR regions decreased or disappeared, which indicated the improvements of system stability. Besides, organized anti-correlation developed between DC3 and AR regions (loop 687-690, H3, loop 722-725, loop 727-730, H4, loop 822-825, H9, H11, loop 888-893, H12). It reflected the opposite movement tendency between these regions and DC3, namely, they moved close to each other along with the simulation. Moreover, distinct correlation also formed between DC3 and some AR regions (H9, loop 843-849, H10). Combining previous study that residues in these regions had low RMSF values, it can be concluded that this correlation patterns decreased residues fluctuation and enhanced the system stability.

## Conclusion

In this work, the three-dimensional structure of cyclopeptide DC3 was firstly constructed by homology modeling technology using 2kux.1.A as template. Then molecular docking was carried out to predict possible binding site and preferred orientation of DC3 into AR. Finally, four systems with best docking score from top four clusters were selected to perform 150 ns all-atom molecular dynamics (MD) simulations. The MM/GBSA method and a series of MD trajectory analyses were subsequently conducted. The analyses of RMSF, binding free energy, DCCM and hydrogen bonds indicated that DC3 showed a higher binding affinity to AR in site 2 (corresponding to H10, H11, loop888-893, H12) and this system showed obvious superiority in stability comparing to other systems. Besides, much more intermolecular hydrogen bonds were constantly formed in system 2 with high occupation. Stronger cross-correlation among DC3 residues and stronger anti-correlation between DC3 and AR residues also exited here. These results suggest that DC3 is most likely to bind to AR in site 2 encompassed by H10, H11, loop888-893, and H12. Subsequently, combining further analysis of free-energy decomposition, interaction surface, distance, and cross-correlation network, it can be observed that hydrogen bonds, hydrophobic, and electrostatic interactions play dominant roles in the recognition and combination of DC3-AR complex. For hydrogen bonds, it frequently existed between K669-H885, D890-R666, S900-T647, and D890-E643. Besides, K665, R666 of DC3, and E706, E709, D890, E893, E897 of AR made great contributions to electrostatic interaction values. V649, V650, V651-V801, and L654, V657, L658-L880, L881 play essential parts of hydrophobic interaction. These results elucidated the detail interaction mechanism of DC3-AR complex and the key residues dominated in specific interaction. These findings will significantly facilitate our understanding of action mode of DC3 to AR at the molecular level, and contribute to the future rational cyclopeptide drug design for prostate cancer.

## Author contributions

JL: conceived and coordinated the study; HZ and TS: did the homology modeling, molecular docking, molecular dynamics simulations, and they wrote the paper; YY and CF: helped to do molecular simulations. All authors analyzed the results and approved the final version of the manuscript.

### Conflict of interest statement

The authors declare that the research was conducted in the absence of any commercial or financial relationships that could be construed as a potential conflict of interest.
